# Unexpected role of *SIX1* variants in craniosynostosis: expanding the phenotype of *SIX1*-related disorders

**DOI:** 10.1136/jmedgenet-2020-107459

**Published:** 2021-01-12

**Authors:** Eduardo Calpena, Maud Wurmser, Simon J McGowan, Rodrigo Atique, Débora R Bertola, Michael L Cunningham, Jonas A Gustafson, David Johnson, Jenny E V Morton, Maria Rita Passos-Bueno, Andrew T Timberlake, Richard P Lifton, Steven A Wall, Stephen R F Twigg, Pascal Maire, Andrew O M Wilkie

**Affiliations:** 1 Clinical Genetics Group, MRC Weatherall Institute of Molecular Medicine, University of Oxford, Oxford, UK; 2 Institut Cochin, INSERM, CNRS, Université de Paris, Paris, France; 3 Centre for Computational Biology, MRC Weatherall Institute of Molecular Medicine, University of Oxford, Oxford, UK; 4 Centro de Estudos do Genoma Humano, Universidade de São Paulo, São Paulo, Brazil; 5 Unidade de Genética Clínica, Instituto da Criança do Hospital das Clínicas, Faculdade de Medicina da Universidade de São Paulo, São Paulo, Brazil; 6 Instituto de Biociências, Universidade de São Paulo, São Paulo, São Paulo, Brazil; 7 Center for Developmental Biology and Regenerative Medicine, Seattle Children's Research Institute, Seattle, Washington, USA; 8 Seattle Children's Craniofacial Center, Seattle Children's Hospital, and Department of Pediatrics, Division of Craniofacial Medicine, University of Washington, Seattle, Washington, USA; 9 Craniofacial Unit, Oxford University Hospitals NHS Foundation Trust, Oxford, UK; 10 West Midlands Regional Clinical Genetics Service and Birmingham Health Partners, Birmingham Women’s and Children’s Hospitals NHS Foundation Trust, Birmingham, UK; 11 Hansjörg Wyss Department of Plastic Surgery, New York University Langone Medical Center, New York, New York, USA; 12 Rockefeller University, New York, New York, USA

**Keywords:** musculoskeletal diseases

## Abstract

**Background:**

Pathogenic heterozygous *SIX1* variants (predominantly missense) occur in branchio-otic syndrome (BOS), but an association with craniosynostosis has not been reported.

**Methods:**

We investigated probands with craniosynostosis of unknown cause using whole exome/genome (n=628) or RNA (n=386) sequencing, and performed targeted resequencing of *SIX1* in 615 additional patients. Expression of SIX1 protein in embryonic cranial sutures was examined in the *Six1* ^*nLacZ/+*^ reporter mouse.

**Results:**

From 1629 unrelated cases with craniosynostosis we identified seven different *SIX1* variants (three missense, including two de novo mutations, and four nonsense, one of which was also present in an affected twin). Compared with population data, enrichment of *SIX1* loss-of-function variants was highly significant (p=0.00003). All individuals with craniosynostosis had sagittal suture fusion; additionally four had bilambdoid synostosis. Associated BOS features were often attenuated; some carrier relatives appeared non-penetrant. SIX1 is expressed in a layer basal to the calvaria, likely corresponding to the dura mater, and in the mid-sagittal mesenchyme.

**Conclusion:**

Craniosynostosis is associated with heterozygous *SIX1* variants, with possible enrichment of loss-of-function variants compared with classical BOS. We recommend screening of *SIX1* in craniosynostosis, particularly when sagittal±lambdoid synostosis and/or any BOS phenotypes are present. These findings highlight the role of *SIX1* in cranial suture homeostasis.

## Introduction

The identification of disease-causing mutations has greatly benefited from the introduction of next-generation sequencing technologies. In particular, whole exome and genome sequencing (WES/WGS) have both accelerated the discovery of new genes involved in rare genetic diseases and, because of their unbiased nature, have enabled expansion of clinical phenotypes associated with known disease genes. Moreover retrospective clinical investigation of the patient and family members can reveal additional, previously unrecognised clinical features.^1^

The initial aim of this work was to investigate a possible genetic cause in a proband affected by speech and language delay, sensorineural hearing loss (HL) and craniosynostosis (CRS), the premature fusion of one or more cranial sutures of the skull. We identified a de novo mutation in *SIX1*, which encodes a homeodomain-containing transcription factor of the *sine oculis* class, originally described in *Drosophila*.^2^ Further analysis of additional unsolved CRS cases using WES/WGS, RNA sequencing or targeted resequencing identified heterozygous variants in *SIX1* in seven further patients with CRS from six unrelated families. Dominantly inherited *SIX1* variants were previously reported in branchio-otic syndrome (BOS; MIM 608389),^3 4^ non-syndromic HL (MIM 605192)^5^ and (rarely) in branchio-oto-renal (BOR) syndrome, associated in addition with renal malformation.^3^ This work uncovers a previously unrecognised role of SIX1 in the maintenance of cranial suture patency.

## Subjects and methods

### Patients

Written informed consent was obtained from all participants/legal guardians. The clinical diagnosis of CRS was confirmed by three-dimensional CT scanning of the skull. When clinically indicated, samples were tested for mutation hotspots in *FGFR2*, *FGFR3*, *TWIST1*, *TCF12* and *ERF*, and significant chromosome aneuploidy was investigated using array comparative genomic hybridisation; samples harbouring mutations known to cause CRS were excluded.

### Genetic analyses

WES or WGS of unrelated probands with CRS of unknown cause (n=103 and n=525 in Oxford and Yale cohorts, respectively) and subsequent bioinformatic analyses were performed as previously described.^6 7^ RNA sequencing of patient osteoblast cell lines (n=386) was previously described.^8^ Targeted screening for *SIX1* variants was performed by resequencing of multiplexed PCR products encompassing the coding regions and intron/exon boundaries of *SIX1* (NM_005982, ENST00000247182) in an additional 615 unsolved patients with CRS (mutation negative for the major known causes). Primer sequences are provided in online supplemental table S1 and the clinical diagnoses of probands screened in online supplemental table S2. Variant calls and coverage information were obtained using amplimap.^9^ Validation and segregation analysis of variants were undertaken by dideoxy-sequencing of PCR products from genomic DNA. Correct sample relationships were confirmed either by comparison of trio-WES data or by analysis of 13 microsatellite markers located on different chromosomes. Previously reported *SIX1* variants were sourced from HGMD Professional V.2019.4, available online communications to the European Society of Human Genetics, and PubMed searches.

10.1136/jmedgenet-2020-107459.supp1 Supplementary data

### *Six1-LacZ* reporter analysis

Heads from heterozygous E18.5 *Six1^nLacZ^* (*Six1^tm1Mair^*) mice^10^ were fixed for 30 min in 4% paraformaldehyde at room temperature and incubated in 15% sucrose overnight at 4°C before embedding in Optimal Cutting Temperature (OCT) compound and freezing in isopentane cooled with liquid nitrogen. One in every 20 frontal sections (10 µm thick) was harvested, incubated in X-gal solution for 2.5 hours at 37°C, mounted on glycerol and imaged with 4× and 10× objectives of an upright fluorescent microscope (Olympus BX63), equipped with an ORCA-Flash4.0 LT Hamamatsu camera, using Metamorph V.7 software.

## Results

### Identification of *SIX1* variants in CRS

**Figure 1:**
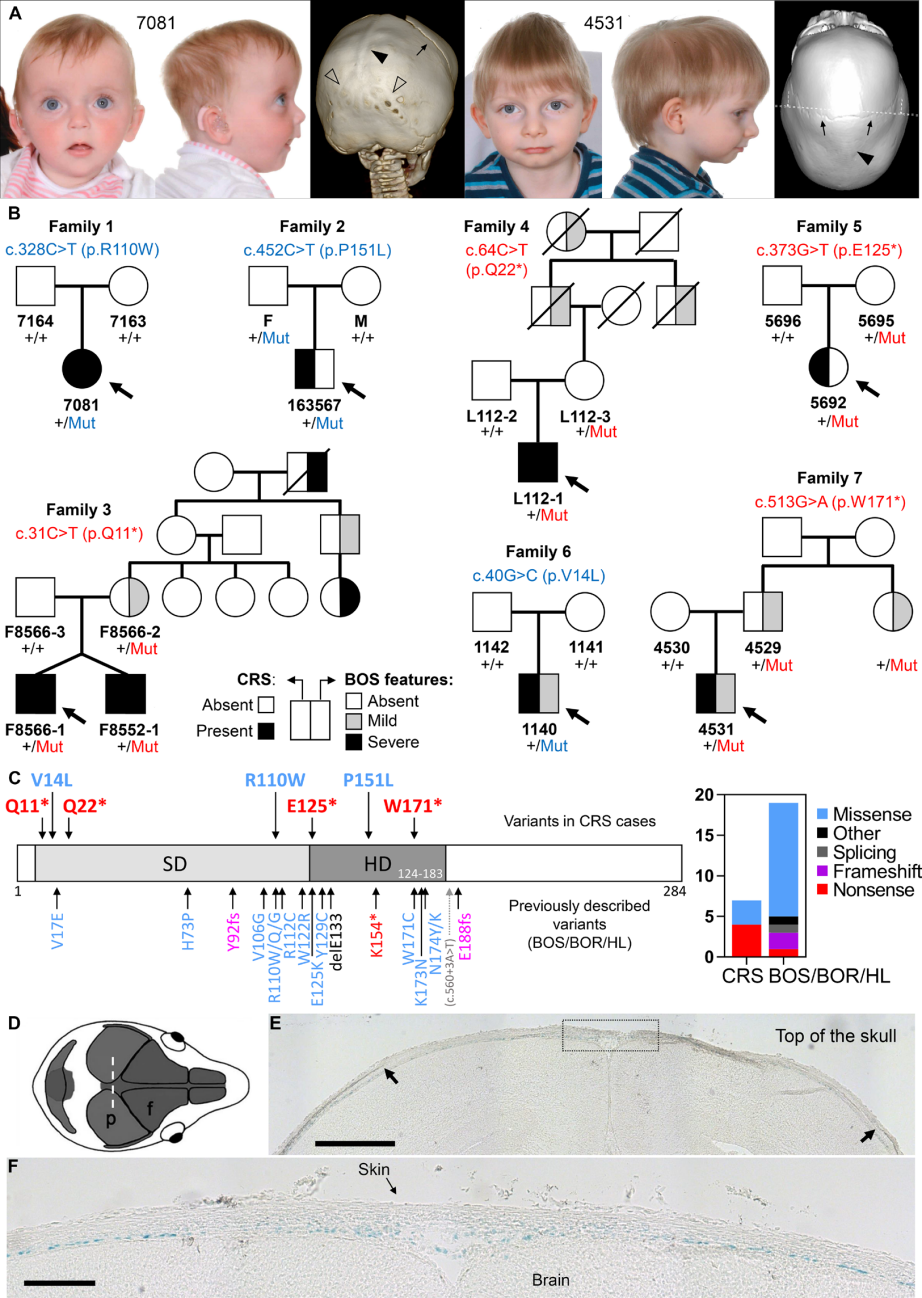
(A) Clinical photographs and CT head scans of individuals 7081 aged 7 months (left) and 4531 aged 2.5 years (right), probands of families 1 and 7, respectively. Note the fused sagittal sutures (filled arrowheads), and in 7081 lambdoid sutures (unfilled arrowheads). The coronal sutures are patent (arrows). (B) Pedigrees of families harbouring missense (blue lettering) or nonsense (red lettering) variants in *SIX1*; individuals affected with CRS shown with black fill (left side of the pedigree symbol) and individuals with BOS features shown with black or grey fill according to severity (right side of the pedigree symbol; mild: preauricular pit or late-onset HL; severe: branchial cyst/fistula or congenital HL). Genotypes are indicated for all available samples. (C) SIX1 variants in congenital disease. (Left) Domain structure of the human SIX1 protein showing the locations of the six domain (SD) and homeodomain (HD). Pathogenic variants, colour-coded according to the key at far right, are shown (above the protein cartoon) for the seven CRS-associated variants identified in this work and (below the cartoon) for previously identified variants in BOS/BOR/HL syndromes. (Right) The chart represents the number of different variants of each pathogenic class identified in CRS or in BOS/BOR/HL. (D–E) SIX1 protein expression revealed by X-gal staining in frontal sections (10 µm thick) of E18.5 *Six1^nLacZ/+^* mouse heads. (D) Representation of the mouse skull from above to illustrate the plane of section (coronal) in the histological sections shown (p, parietal bone; f, frontal bone). (E) Arrows indicate the superior margins of the parietal bones, and the dotted box (enlarged in a nearby section in (F) shows the sagittal suture. Blue nuclei (X-gal positive) indicate SIX1 expression. Scale bars: 500 µm (E) and 150 µm (F). BOR, branchio-oto-renal; BOS, branchio-otic syndrome; CRS, craniosynostosis; HL, hearing loss.

Parent–child trio-based WES was performed on proband 7081, affected by sagittal and bilambdoid CRS (figure 1A, left), bilateral sensorineural HL, and speech and language delay. This identified a de novo *SIX1* c.328C>T (p.R110W) mutation (family 1 in figure 1B, table 1). *SIX1* variants, including p.R110W, were previously reported in individuals with BOS/BOR syndromes (dominantly inherited disorders characterised by variable hearing impairment, preauricular pits, branchial defects±kidney malformations) or non-syndromic HL (figure 1C).^3–5^ No additional candidate variants were identified to explain the CRS in proband 7081, who retrospectively was noted to manifest additional features of BOS, including bilateral preauricular skin tags, ear pits and a possible sinus over the right sternocleidomastoid (see online supplemental case report).

**Table 1:** *SIX1* variants identified in subjects with CRS

Family	1	2	3		4	5	6	7
ID	7081	163567	F8566-1 (twin 1)	F8552-1 (twin 2)	L112-1	5692	1140	4531
*SIX1* variant	c.328C>T (p.R110W)	c.452C>T (p.P151L)	c.31C>T (p.Q11*)	c.31C>T (p.Q11*)	c.64C>T (p.Q22*)	c.373G>T (p.E125*)	c.40G>C (p.V14L)	c.513G>A (p.W171*)
Variant type (CADD score)*	Missense (33)	Missense (32)	Nonsense (38)	Nonsense (38)	Nonsense (37)	Nonsense (38)	Missense (24.4)	Nonsense (39)
Previously reported?	Recurrent in BOS/BOR	Novel†	Novel†	Novel†	Novel†	Novel†	Novel†	Novel†
Inheritance	De novo	Inherited (paternal)	Inherited (maternal)	Inherited (maternal)	Inherited (maternal)	Inherited (maternal)	De novo	Inherited (paternal)
Sutures affected								
Coronal (L, R)				+, +				
Sagittal	+	+	+	+	+	+	+	+
Metopic								
Lambdoid (L, R)	+, +		+, +		+, +	+, +		
Syndromic features	Speech/language delay, ear pits/tags, unilateral neck sinus, sensorineural hearing loss.	No (borderline short stature, mild anteverted nares).	Branchial fistula.	Branchial fistula, restricted growth, posterior urethral valves.	Mild conductive hearing loss, unilateral branchial cyst/fistula.	No.	No (preauricular pits only).	No (occult bilateral branchial cysts).
Family history	Not applicable.	Not documented.	Branchial cysts, preauricular pit (maternal branch).		Hearing loss (maternal branch).	No.	Not applicable.	Moderate hearing loss (paternal branch).

*Combined Annotation Dependent Depletion score (CADD GRCh38-V.1.6).

†None of the novel variants is listed in gnomAD V.2.1.1 (minimum coverage ~245 000 alleles).^15^

BOR, branchio-oto-renal; BOS, branchio-otic syndrome; CRS, craniosynostosis; ID, identifier; L, left; R, right.

To determine whether CRS was causally associated with the *SIX1* mutation, we interrogated existing cohorts of genetically undiagnosed CRS investigated by either WES/WGS (n=628)^6 7^ or RNA sequencing (n=386).^8^ This highlighted four additional families carrying rare heterozygous *SIX1* variants (families 2–5; figure 1B, table 1). No additional candidate variants explained the CRS in these cases. To investigate further the significance of *SIX1* variants, we performed targeted resequencing of *SIX1* in a cohort of 615 additional patients with any type of CRS, leading to the discovery of two further families with heterozygous *SIX1* variants (families 6 and 7; figure 1B, table 1). In addition to the initial finding (family 1), one of the variants from the cohort screen (c.40G>C; p.V14L) arose de novo in the proband (family 6), whereas in the remaining five families (families 2–5 and 7) the variant had been transmitted from one of the parents. Dideoxy-sequence verification of all *SIX1* variants is shown in online supplemental figure S1. Of 1629 probands analysed, *SIX1* variants were present in 3 of 822 (0.4%) sagittal synostosis, 4 of 112 (3.6%) multisuture (excluding bicoronal or bilambdoid) synostosis and 0 of 695 with other types of suture fusion (online supplemental table S2).

### Phenotype and genotype–phenotype correlation

Overall we identified eight patients with CRS heterozygous for variants in *SIX1*, from seven unrelated families. The variants comprised de novo missense (n=2) or inherited nonsense (n=4) or missense (n=1) changes (figure 1B, table 1). The missense changes are located at residues conserved in all six human SIX paralogues, as well as *Drosophila sine oculis* (online supplemental box S1); Combined Annotation Dependent Depletion (CADD) scores are correspondingly high (24.4–33). Interestingly, all individuals with CRS had sagittal synostosis (figure 1A), in five instances combined with fusion of both coronal (n=1) or both lambdoid (n=4) sutures (table 1). Six of these eight individuals (75%) presented additional clinical features compatible with BOS (detailed descriptions in the online supplemental case reports), notably branchial arch defects (preauricular pits or tags, neck swellings or sinuses, n=5) and/or HL (n=2). No symptomatic renal abnormalities were documented, consistent with a low incidence of renal disease caused by *SIX1* variants in previous reports^4^; however targeted renal imaging was undertaken in only three patients. In three of the inherited cases, retrospective analysis indicated the presence of BOS-related features in additional family members and apparent non-penetrant carrier status in others (figure 1B, table 1).

Considering the predicted functional consequences of SIX1 variants, four of the seven different variants (57%) are stop-gain, representing a 2.7-fold enrichment of loss-of-function (LoF) variants in CRS compared with the spectrum previously reported in BOS/BOR/HL, which are more often caused by missense variants (73.7% of the 19 different pathogenic variants identified in 27 unrelated families reported in the literature; figure 1C, online supplemental table S3). Although this difference is not significant (p=0.15, two-tailed Fisher’s exact test), there is apparent enrichment (p=0.01) when only the nonsense variants are considered (4 of 7 in CRS vs 1 of 19 in BOS/BOR/HL).

### SIX1 expression in the sagittal suture

To investigate whether SIX1 is expressed in cranial sutures, we examined a previously reported transgenic mouse line with the nls-*LacZ* reporter inserted into the first exon of *Six1*.^10^ X-gal staining of frontal sections of E18.5 *Six1^nLacZ/+^* embryonic heads demonstrated β-galactosidase activity in a layer basal to the growing bones, likely corresponding to the dura mater, and extending into the mesenchyme of the future sagittal suture (figure 1D–F). By contrast, no significant staining was observed in the osteogenic fronts or mid-sutural mesenchyme of the coronal sutures (online supplemental figure S2).

## Discussion

This study identifies enrichment of heterozygous deleterious *SIX1* variants in patients with CRS, expands the phenotype of *SIX1*-related disorders and provides evidence for a previously unrecognised role of SIX1 in normal homeostasis of the cranial sutures.

Of note, sagittal suture fusion was present in all eight affected individuals, an unusual pattern as monogenic types of CRS more commonly involve the coronal sutures.^11^ In four subjects, the sagittal synostosis was present together with the involvement of both lambdoid sutures (‘Mercedes-Benz’ pattern), a rare combination of suture fusions present in 23 of the 1629 probands screened for *SIX1* mutations (online supplemental table S2). In a cohort of 4250 unselected CRS cases treated in a single department, only 39 patients were diagnosed with combined sagittal and bilambdoid synostosis^12^; our finding that 4 of 8 patients with *SIX1* pathogenic variants exhibited the Mercedes-Benz pattern represents a significant enrichment (p<10^−6^, two-tailed Fisher’s exact test).

In addition to CRS, six of the eight patients exhibited additional clinical features compatible with *SIX1*-related BOS/BOR/HL. However, the associated anomalies were sometimes minor (such as ear pits) and were retrospectively identified in some cases. Significant HL was documented in only two of eight individuals with CRS. Whereas de novo missense mutations accounted for two sporadic cases, parental transmission occurred in the other five families, and in three of these an extended family history compatible with BOS±isolated HL was evident (figure 1B). Although intrafamilial phenotypic variability is frequent in *SIX1*-related disease, non-penetrance is uncommon^3 4^; by contrast, non-penetrance occurred in all five families showing parental transmission of the variant, including three confirmed *SIX1*-heterozygous individuals (figure 1B, families 2, 4 and 5). Notably, in four of these families, the *SIX1* variant encodes a nonsense change, only previously described once in BOS/BOR/HL, in a patient additionally noted to have macrocephaly.^13^

In summary, whereas the classic BOS/BOR/HL-associated variants are usually missense substitutions that may have dominant-negative activity (figure 1C),^4 14^ we propose that heterozygous *SIX1* LoF variants are associated with a haploinsufficiency phenotype that overlaps with BOS/BOR/HL, but includes a propensity to CRS in some individuals and non-penetrance in others. According to the gnomAD (V.2.1.1) database,^15^ *SIX1* is moderately constrained (probability of LoF intolerance(pLI)=0.65; observed/expected=0.17 (0.07–0.52) for LoF alleles), with 10 LoF alleles observed in a minimum of 238 186 alleles surveyed. Although this supports that halving the effective *SIX1* dosage sometimes has mild consequences, the enrichment of CRS associated with *SIX1* LoF alleles (4 of 3258) is highly significant (p=0.00003, Fisher’s exact test).

SIX1 is a homeodomain-containing transcription factor that is essential for normal development of several organs. In the head, *Six1* is expressed in different lineages in avian and murine embryos, and *Six1*-deficient mice display severe craniofacial malformations during embryonic development.^16 17^ To our knowledge, however, *Six1* expression has not been formally analysed in the cranial sutures. Considering the specificity of the sagittal suture involvement in all the *SIX1*-positive cases, we focused our analysis on this suture and used a nls-*LacZ* reporter gene (inserted at the *Six1 locus*)^10^ to mirror the spatiotemporal pattern of SIX1 expression in heterozygous mouse embryos (E18.5). The presence of SIX1 associated with a basal layer between the growing bone and the brain is intriguing. This layer most likely represents the dura mater, which was recently shown in a single cell transcriptomic analysis to be enriched for *Six1* expression.^18^ Paracrine signals from the dura mater contribute to maintenance of suture patency and closure,^19^ by coordinating availability of secreted factors such as fibroblast growth factors and β-type transforming growth factors, signalling pathways potentially regulated by SIX1.^20^ However, to our knowledge, no genetic mutants have been shown to act primarily by disturbing the dura mater–suture interactions. This work therefore provides a starting point to investigate the contribution of SIX1 dosage to suture homeostasis and the downstream targets perturbed in the presence of pathogenic variants.

In summary, both missense and nonsense variants in *SIX1* confer a substantially increased risk of CRS. We recommend testing for *SIX1* variants in undiagnosed CRS with sagittal involvement, especially when occurring in combination with lambdoid synostosis and/or associated with BOS/BOR/HL-related clinical features in the proband or extended family.
