# Supplementary material for: Unexpected role of SIX1 variants in craniosynostosis: expanding the phenotype of SIX1-related disorders
Source: J Med Genet. 2021 Jan 12;59(2):165–9. doi: 10.1136/jmedgenet-2020-107459 (PMC8273188; doi:10.1136/jmedgenet-2020-107459)
Supplement: Supplementary data [file jmedgenet-2020-107459supp001.pdf]

Supplementary Information

	Page
Supplementary case reports	1
Table S1. Primer sequences and methods used for PCR amplification	8
Table S2. Phenotypic composition of cohorts of unsolved CRS probands	9
Table S3. Previously described *SIX1* variants in BOS/BOR/HL	10
Box S1. Protein sequence alignments of all human SIX-family paralogues and *Drosophila melanogaster* sine oculis (SO)	12
Figure S1. Dideoxy-sequence verification of *SIX1* variants	13
Figure S2. SIX1 expression revealed by X-gal staining in additional coronal section of E18.5 $Six1^{nLacZ/+}$ mouse head	14
References	15

Supplementary case reports

Family 1: *SIX1* c.328C>T (p.R110W)

This girl (ID 7081) is the fourth child of unrelated parents. The father had attended a special needs school and had required a heart transplant aged 10 years (yr) owing to cardiomyopathy of unknown cause. The pregnancy was uncomplicated. She was born at term +12 days by normal vaginal delivery, having presented in occipito-posterior position. Bilateral hearing loss (HL) was detected on neonatal screening and bilateral hearing aids were fitted.

On paediatric review at the age of 5 months (mo) she was noted to have wide frontal bossing, with a patent anterior fontanelle, sagittal ridge and constricted occiput with inferior central flattening. She had noisy breathing attributed to tracheomalacia. Craniofacial assessment at the age of 8 mo confirmed frontal bossing with a patent anterior fontanelle. Her occipito-frontal circumference (OFC) was 44.5 cm (+0.6 SD) and cephalic index (CI) was 0.79 (normal range in girls 0.76-0.84). A computed tomography (CT) scan with venogram demonstrated sagittal and partial lambdoid synostosis with normal cerebral venous anatomy and no evidence of Chiari malformation. She underwent total calvarial remodelling at the age of 1 yr 5 mo. Diagnostic laboratory genetic testing, which included sequencing of *ERF*, *GJB2* and *TWIST1*, mutation hotspots in *FGFR1*, *FGFR2*, *FGFR3* and *GJB6*, and MLPA analysis including *TWIST1*, was normal.

Formal hearing assessment by play audiometry at the age of 2 yr 9 mo confirmed moderate HL bilaterally (50-60 dB HL soundfield and 20-50 dB HL bone conduction), indicating a predominantly sensorineural component. Echocardiography showed normal left ventricular function. Speech and language assessment at the age of 3 yr 3 mo showed

Calpena E, *et al. J Med Genet* 2022; 59:165–169. doi: 10.1136/jmedgenet-2020-107459

significantly delayed core language and speech skills (CELF Preschool – 2 UK scores 0 [sentence structure], 2 [word structure] and 4 [expressive vocabulary]; normal range for each 7-13). Developmental assessment (Schedule of Growing Skills II) at the age of 5 yr 0 mo showed a cognitive attainment of 4 yr 0 mo.

On further review at the age of 5 yr, following identification of the *SIX1* mutation, she was noted to have bilateral preauricular skin tags, one pit over the left ear and two over the right, and a possible sinus over the inferior border of her right sternocleidomastoid; this had been present since birth and occasionally discharged clear fluid. Ultrasound scanning of her kidneys was normal.

The *SIX1* c.328C>T (p.R110W) variant was identified as a *de novo* mutation by parent-child trio-based exome sequencing (ES) (Oxford ES/GS cohort). This variant has previously been reported in at least seven unrelated families with branchio-otic syndrome (BOS) or non-syndromic HL,[1-4] and additional variants affecting the same reside (p.R110G, p.R110Q) have been described in these disorders.[1,5] The R110W variant has been subject to extensive functional characterization.[6,7]

Two additional *de novo* variants were identified by ES, but were considered unlikely to be clinically significant. These were NM_001041:c.2244+2T>A in *SI* (sucrase-isomaltase) (absent in gnomAD; pLI = 0); and NM_024757:c.3462-41C>T in *EHMT1* (rs376124897: 3 alleles in gnomAD, frequency 0.00002638 in European [non-Finnish]; pLI = 1). Biallelic mutations in *SI* cause sucrose-isomaltase deficiency, however no additional *SI* variant was evident in the proband's exome sequence. Monoallelic loss-of-function mutations of *EHMT1* cause Kleefstra syndrome, but the variant had no predicted effect on splicing (NNSPLICE 0.9)[8] and was considered to be too frequent in the background population.

Family 2: *SIX1* c.452C>T (p.P151L)

This boy (ID 163567) is the fifth child born to unrelated parents. There was no family history of abnormal head shapes or craniosynostosis. With the exception of a paternal first cousin born with an open neural tube defect, there was no family history of structural birth defects.

He was born at 36 weeks' gestation with a birthweight of 3118 grams (+1.1 SD) and was noted at birth to have a scaphocepahlic head shape, without other congenital anomalies. Preoperative anthropometric measurements are not available. A preoperative CT scan at the age of 3 mo demonstrated isolated sagittal craniosynostosis; additional features present were a midline cleft of the mandibular symphysis, a previously-erupted left mandibular central incisor, and symmetrically paired, narrow linear parasagittal defects extending bidirectionally from the lambdoid sutures into the adjoining parietal and occipital bones. He was treated with a modified pi procedure at 13 weeks of age at an outside institution.

Calpena E, *et al. J Med Genet* 2022; 59:165–169. doi: 10.1136/jmedgenet-2020-107459

He was referred for further assessment at 1 yr of age owing to persistent scaphocephaly and residual skull defects. His height was below the 1st centile (-2.8 SD), his weight at the 25th centile and his OFC at the 75th-90th centile. His physical examination was otherwise normal except for mild brachydactyly and anteverted nares. He had normal neurocognitive and motor development with precocious language skills. A repeat cranioplasty was performed at this stage.

He was enrolled prospectively in an RNA sequencing project focused on children born with isolated, non-syndromic single suture craniosynostosis. A *SIX1* c.452C>T (p.P151L) variant was identified, confirmed on Sanger sequencing, and demonstrated to have been paternally inherited. The father was healthy, with normal hearing, but commented that his left ear was lower than his right, which had caused difficulty with wearing spectacles. There was no other relevant family history. The Pro151 residue is located at the 28th position of the 60-amino acid homeodomain of SIX1, corresponding to the start of α-helix II. This proline is conserved in all six human SIX paralogues, as well as *Drosophila sine oculis* (Supplementary Box 1), and no variants at this position are listed in the gnomAD database (v2.1.1, >251,000 alleles surveyed).

Family 3: *SIX1* c.31C>T (p.Q11*)

The proband (twin 1; ID F8566-1) and his dizygotic twin brother (twin 2; ID F8552-1) are the only children of non-consanguineous parents. The family history revealed the presence of a preauricular pit in the mother and branchial cyst in her maternal grandfather and in a maternal cousin.

The twins were delivered preterm (36 weeks' gestation) by caesarean section, in good condition. The pregnancy was uncomplicated except that the mother reported light vaginal bleeding during the early months. The birth weights of the first and second-born twins were 2360 g and 1752 g, and birth lengths were 42 cm and 39 cm, respectively. Physical examination revealed a branchial fistula in the neck of each twin. Apart from phototherapy for jaundice, both twins remained well during an initial 20-day hospital stay to achieve satisfactory weight gain.

At the age of 5 mo, the twins were referred for neurosurgical evaluation because of abnormal configuration of the skull. Cranial CT scan with 3D reconstruction showed premature fusion of the sagittal and part of the lambdoid sutures in twin 1 and fusion of the sagittal and both coronal sutures in twin 2. Surgical correction of the craniosynostosis was performed at the age of 11 mo in both twins. Twin 2 was diagnosed with posterior urethral valves and treated at the age of 2 yr 8 mo by an endoscopic procedure. In addition the branchial fistula was surgically removed in each twin.

Calpena E, *et al. J Med Genet* 2022; 59:165–169. doi: 10.1136/jmedgenet-2020-107459

Both twins were referred for endocrinological assessment, initially at the age of 3 yr, because of poor weight gain and growth. Aged 4 yr 3 mo their weights were 13.2 kg (-2.24 SD) and 11.5 kg (-3.7 SD), heights 91.5 cm (-2.9 SD) and 88 cm (-3.7 SD), and OFCs 51 cm (+0.3 SD) and 49 cm (-1.1 SD) in twins 1 and 2, respectively. Both boys had a similar triangular-shaped face, with retrognathia, prominent ears and a right-sided scar in the neck and both had bilateral clinodactyly of the 5th fingers. They had normal developmental milestones and were performing well at a regular school at the age of 7 yr.

Additional investigations included brainstem auditory evoked responses, which were abnormal in the right ear in twin 2, and echocardiogram and abdominal ultrasound, which were normal in both twins. The 2nd twin, who exhibited more impaired growth, was started on growth hormone treatment at the age of 6 yr.

Genetic investigation using ES did not reveal any rare variant in a known gene associated with craniosynostosis, but identified a heterozygous variant in *SIX1*, c.31C>T (p.Q11*), in twin 2 (this region was covered by only one normal read in twin 1). The variant was confirmed by dideoxy-sequencing in both twins and in their mother, who presented with a preauricular pit only.

Family 4: *SIX1* c.64C>T (p.Q22*)

The male proband (ID L112-1) was born full-term to healthy parents after an uncomplicated pregnancy. An abnormal head shape was noted by the child's paediatrician at age 2 yr; a prominent mastoid bulge on the right side prompted a CT scan at this time, demonstrating sagittal and bilateral lambdoid synostosis. The patient underwent calvarial vault remodelling just before age 3 yr. At age 4 yr, the child underwent formal hearing assessment by an audiologist who identified conductive HL in the left ear. Both the family and paediatrician reported normal neurodevelopment at this time, however no formal neurocognitive testing was performed. The proband's only other medical condition is a branchial cyst with fistulization to the medial neck that periodically becomes inflamed and drains purulent material, which has been managed with antibiotic therapy to date. No renal ultrasound has been performed.

At age 4 yr, the parents enrolled their family in a genetic study of lambdoid craniosynostosis through Yale University. The case-parent trio underwent exome sequencing, which did not reveal any exonic *de novo* variants in the proband, rare recessive genotypes, or pathogenic variants in known craniosynostosis genes. Further evaluation for transmitted damaging variants identified a maternally inherited nonsense mutation in *SIX1* (c.64C>T; p.Q22*). While the parents are unaware of any other craniosynostosis in their family, the maternal family history includes at least 3 individuals with non-syndromic HL (figure 1B).

Calpena E, *et al. J Med Genet* 2022; 59:165–169. doi: 10.1136/jmedgenet-2020-107459

Family 5: *SIX1* c.373G>T (p.E125*)

This girl (ID 5692) has healthy non-consanguineous Asian parents. There is no family history of renal problems, deafness or congenital abnormality. She presented with an unusual head shape, which was shown by CT scanning to be caused by a combination of sagittal and bilambdoid synostosis and was associated with a type 1 Chiari malformation. Additional clinical features were long, down-slanting palpebral fissures, prominent eyes and an apparent epibulbar dermoid of the right eye. A diagnosis of Crouzon syndrome was considered, but genetic investigations (chromosome microarray, *FGFR2*, *FGFR3*, *IL11RA*, *TWIST1*, *ERF*) were normal.

She was further investigated by parent-child trio-based ES (Oxford ES/GS cohort). *De novo* and recessive disease models of causation were first considered, however no plausible candidate variants were identified. A potentially pathogenic *SIX1* c.373G>T (p.E125*) nonsense change was noted, however this was originally classified as a variant of unknown significance because (i) it was maternally inherited (ii) there was no history of HL or other features of BOS and (iii) no association of *SIX1* variants with craniosynostosis was reported at the time. This interpretation was revised following the identification of other similarly affected patients presented in this work.

The parents failed to attend for follow up after the identification of the *SIX1* variant, so it has not been possible to re-examine the child or her mother, or to arrange further investigations in the light of the genetic findings.

Family 6: *SIX1* c.40G>C (p.V14L)

This boy (ID 1140) was the first child of unrelated parents. There was no significant family history. During pregnancy the fetal head was reported to have engaged early. The delivery was normal and postnatally, growth and development were satisfactory, but an elongated head shape was noted at the age of 7 mo. No hearing problems were recorded.

On formal assessment at the age of 2 yr 4 mo, his OFC was 54.0 cm (+3.4 SD) and CI was 0.67 (normal range in boys 0.76-0.83). He was scaphocephalic, with a high bulging forehead, mild temporal pinching, and prominent occiput. There were no dysmorphic features, no neck swelling and the ears were morphologically normal apart from bilateral pre-auricular pits. CT head scan with bone windows demonstrated sagittal synostosis with copper-beating on the right posteriorly. Formal hearing assessment (free field pure tone audiometry) was within normal limits (15 dB HL bilaterally).

Total calvarial remodelling was performed at the age of 2 yr 7 mo, without complications. On pre-operative neuropsychological assessment (Bayley Scales of Infant Development – 2nd Edition) his Mental Development Index was 89 (low average). On the

Griffiths Mental Development Scales his attainment was 2 yr 6 mo (eye-hand coordination), 2 yr 10 mo (personal-social) and 3 yr (locomotor). There were no post-operative problems and he was lost to follow-up when his family emigrated 2 months post-operatively. Extensive research laboratory-based genetic screening, which included *ERF*, *IL11RA*, *SMAD6*, *TCF12*, *TWIST1* and mutation hotspots in the *FGFR1, FGFR2* and *FGFR3* genes, was normal.

The *SIX1* c.40G>C (p.V14L) variant was identified in the targeted resequencing panel. The corresponding sequence was normal in both parental samples (relationships confirmed by analysis of 13 microsatellite markers), demonstrating that the variant had arisen as a *de novo* mutation. The mutated Val14 residue locates in the α-helix of the Six domain (SD) and is conserved in all six human SIX-family paralogues and *Drosophila sine oculis* (Supplementary Box 1). No variants at the Val14 residue are listed in the gnomAD database (v2.1.1, >246,000 alleles surveyed), nor have variants previously been described in any of BOS, HL or BOR (branchio-oto-renal syndrome). Val14 locates close to residue Val17,[9] mutated in BOS (c.50T>A, p.V17E),[1] which has been the subject of extensive functional characterization.[6,7,9]

Family 7: *SIX1* c.513G>A (p.W171*)

This boy (ID 4531) is the second son of unrelated parents. There was no family history of unusual head shapes but, retrospectively, there was a family history of HL in the father and paternal aunt, as described below.

Although an elongated head shape was noticed at birth, this was not investigated at the time. At the age of 2 yr 2 mo, during a hospital admission for a febrile convulsion, the head shape was again noted and a CT scan demonstrated sagittal synostosis. On craniofacial assessment at the age of 2 yr 6 mo, his OFC was 47 cm (-1.5 SD) and CI 0.72; scaphocephaly with moderate frontal bossing and an occipital bullet were confirmed. The parents mentioned that they had noticed lumps in the neck bilaterally, but were reassured. He proceeded to have subtotal calvarial remodelling at the age of 2 yr 11 mo. At this time, pure tone audiometric screening down to 20 dB HL was normal. Formal neuropsychological assessment using the Wechsler Pre-School and Primary Scales of Intelligence (2003) and motor subscales of the Bayley Infant and Toddler Development Scales (2006) showed average results; a speech and language assessment was also within the normal range. He has continued to make good progress, with no concerns regarding development or schooling up to the age of 11 yr.

The *SIX1* c.513G>A (p.W171*) variant was identified in targeted resequencing panel, and demonstrated to have been paternally inherited. On further questioning, the 47 yr old father stated that he had worn hearing aids for the previous three years; his HL had been attributed to significant noise exposure as a young man, both at work and recreationally. The

father's sister aged 45 yr, who was also heterozygous for the *SIX1* variant, had noticed reduced hearing over several years and on audiological testing was found to have bilateral mild-moderate sensorineural HL (20 dB HL at 250 Hz increasing to 45 dB HL at 8 kHz, for both air and bone conduction).

Table S1. Primer sequences and methods used for PCR amplification

Primers	Sequence (5'-3')
SIX1-Ex1.1-F	ACACTGACGACATGGTTCTACACACCGCCAAGTTCCGACTCCG
SIX1-Ex1.1-R	TACGGTAGCAGAGACTTGGTCTCTTCTCGGCCTCCACGTAATGC
SIX1-Ex1.2-F	ACACTGACGACATGGTTCTACACAGTTCTCGCCTCACAACCACC
SIX1-Ex1.2-R	TACGGTAGCAGAGACTTGGTCTTTCCTAGGGTCGCCCGAGTCG
SIX1-Ex2-F	ACACTGACGACATGGTTCTACAGTTGGTGACAGATTGCACAATGGC
SIX1-Ex2-R	TACGGTAGCAGAGACTTGGTCTTGCTGCTCCAGGAATCCCTTCG

Primer pairs were designed to amplify the coding regions of the two exons and the intron/exon boundaries of *SIX1* (NM_005982.4, ENST00000247182). Universal CS1 (5′-ACACTGACGACATGGTTCTACA-3′) and CS2 (5′-TACGGTAGCAGAGACTTGGTCT-3′) adaptor sequences were included at the 5′ ends of all target-specific forward and reverse primers, respectively. Target regions were amplified using multiplexed PCR (Ex1.1 in mix A, Ex1.2 and Ex2 in mix B), from 20 ng of genomic DNA using FastStart Taq DNA Polymerase (Roche) and the following cycling conditions: 8 min denaturation step at 95°C, followed by 35 cycles of 95°C for 30 s, 65°C for 30 s and 72°C for 35 s, and a final extension step of 72°C for 7 min. For each patient, the multiplexed PCR products were combined, diluted 100-fold and used in a second 9-cycle PCR reaction, designed to incorporate Illumina sequence-specific adaptors and sample indexes (Fluidigm) and using the Q5 High-Fidelity DNA Polymerase (NEB). The indexed PCR products were pooled, and the library was purified and sequenced using MiSeq 500 or 600 cycle reagent kits on the MiSeq platform (Illumina) as previously described.[10] The deleteriousness of identified variants was predicted using CADD scores (CADD GRCh38-v1.6).[11]

Calpena E, *et al. J Med Genet* 2022; 59:165–169. doi: 10.1136/jmedgenet-2020-107459

Table S2. Phenotypic composition of cohorts of unsolved CRS probands

	Oxford WES/WGS cohort		Yale WES cohort		Seattle RNA sequencing cohort		Resequencing cohort		Total	
**Phenotype[a]**	***SIX1*-negative**	***SIX1*-positive**	***SIX1*-negative**	***SIX1*-positive**	***SIX1*-negative**	***SIX1*-positive**	***SIX1*-negative**	***SIX1*-positive**	***SIX1*-negative**	***SIX1*-positive**
NS bicoronal	22						4		26	
NS multiple (sagittal+bilambdoid)	2	1	4				5		11	1
NS multiple (other)	10		12				25		47	
NS sagittal	2		301		194	1	275	2	772	3
NS metopic			169		93		43		305	
NS bilambdoid			2				2		4	
NS unicoronal	5				78		147		230	
NS unilambdoid			19		20		5		44	
S bicoronal	5						8		13	
S bilambdoid							1		1	
S metopic	5		5				18		28	
S multiple (sagittal+bilambdoid)	2	2	1	1			5		8	3
S multiple (other)	24		4				14		42	
S sagittal	9		6				32		47	
S unilambdoid			1				2		3	
S unicoronal	6						11		17	
Carpenter	3								3	
craniofrontonasal syndrome							1		1	
Crouzon							5		5	
Pfeiffer							2		2	
Saethre-Chotzen	5						4		9	
Other							4		4	
**Total**	**103**		**525**		**386**		**615**		**1629**	

[a]Abbreviations: NS, non-syndromic; S, syndromic

Table S3. Previously described *SIX1* variants in BOS/BOR/HL phenotypes[a]

cDNA substitution	Amino acid substitution	Phenotype	References	Variant type	Location	Additional information	gnomAD (v2.1.1)
c.50T>A	p.V17E	BOS	[1,6,9]	missense	SD		
c.218A>C	p.H73P	BOS	[1,9]	missense	SD		
c.273_274insC	p.Y92Lfs*62	BOS	[12]	frameshift (LoF)	(SD)		
c.317T>G	p.V106G	BOS	[1,6,9]	missense	SD		
c.329G>A	p.R110Q	BOS	[1,9]	missense	SD		
c.328C>G	p.R110G	BOR	[5]	missense	SD		
c.328C>T	p.R110W	BOS/non-syndromic HL	[2-4,6]	missense	SD	Recurrent (reported in 7 cases/families)	
c.334C>T	p.R112C	BOS	[1,6,9]	missense	SD		
c.364T>A	p.W122R	BOS	[13]	missense	SD		
c.373G>A	p.E125K	HL and preauricular pits	[14]	missense	HD		
c.386A>G	p.Y129C	BOS	[3,6,15,16]	missense	HD	Recurrent (reported in 3 cases/families)	Total MAF 0.000003977 (1 allele)
c.397_399del	p.E133del	BOR / non-syndromic HL	[3,6]	aa deletion (other)	HD		
c.460A>T	p.K154*	HL (+macrocephaly)	[17]	nonsense (LoF)	(HD)		
c.513G>C	p.W171C	HL	[18]	missense	HD		
c.519G>C	p.K173N	BOS	[19]	missense	HD		
c.520A>T	p.N174Y	HL	[20]	missense	HD		
c.522C>G	p.N174K	HL	[21]	missense	HD		
c.560+3A>T		BOS	[16]	splicing (LoF)			
c.561_564del	p.E188Tfs*63	BOS	[22]	frameshift (LoF)			

Additional previously described variants. Unlikely to be causative							
**cDNA substitution**	**Amino acid substitution**	**Phenotype**	**Reference**	**Variant type**	**Location**	**Additional information**	**gnomAD (v2.1.1)**
c.401A>G	p.K134R	Conotruncal heart defects	[23]	missense	HD	Arg in the equivalent position of human SIX3/5/6	
c.679G>T	p.D227Y	Renal hypodysplasia	[24]	missense		Too common	Total MAF 0.0001069 (30 alleles); European non-Finnish 0.0002360 (30 alleles)
c.746C>T	p.P249L	BOR	[16]	missense		Too common	Total MAF 0.0001523 (43 alleles); Ashkenazi Jewish 0.003087 (32 alleles)

[a]Abbreviations: BOS, branchio-otic syndrome; BOR, brachio-oto-renal syndrome; HL, hearing loss

Calpena E, *et al. J Med Genet* 2022; 59:165–169. doi: 10.1136/jmedgenet-2020-107459

Box S1. Protein sequence alignments of all human SIX-family paralogues and *Drosophila melanogaster* sine oculis (SO)

```
                                                                       V14L    V17E
                                                                          \    /
sp|Q15475|SIX1_HUMAN            ------------------------------MSMLPSFGFTQEQVACVCEVLQQGGNLERL        30
sp|Q9NPC8|SIX2_HUMAN            ------------------------------MSMLPTFGFTQEQVACVCEVLQQGGNIERL        30
sp|O95343|SIX3_HUMAN            GAGGGGGGGSRAP-----P-------EELSMFQLPTLNFSPEQVASVCETLEETGDIERL        108
sp|Q9UIU6|SIX4_HUMAN            GAVAAAAAGAAADQVQLHSELLGRHHHAAAAAAQTPLAFSPDHVACVCEALQQGGNLDRL        129
sp|Q8N196|SIX5_HUMAN            GAAAAGAEGPGSPGVP------G-SPPEAASEPPTGLRFSPEQVACVCEALLQAGHAGRL        107
sp|O95475|SIX6_HUMAN            ------------------------------MFQLPILNFSPQQVAGVCETLEESGDVERL        30
sp|Q27350|SO_DROME              GVVSGGGS--------------------GGRENLPSFGFTQEQVACVCEVLQQAGNIERL        124
                                                                    : *: ::** ***.* : *.  **
                                                                             H73P
                                                                              |
sp|Q15475|SIX1_HUMAN            GRFLWSLP----ACDHLHKNESVLKAKAVVAFHRGNFRELYKILESHQFSPHNHPKLQQL        86
sp|Q9NPC8|SIX2_HUMAN            GRFLWSLP----ACEHLHKNESVLKAKAVVAFHRGNFRELYKILESHQFSPHNHAKLQQL        86
sp|O95343|SIX3_HUMAN            GRFLWSLPVAPGACEAINKHESILRARAVVAFHTGNFRDLYHILENHKFTKESHGKLQAM        168
sp|Q9UIU6|SIX4_HUMAN            ARFLWSLP----QSDLLRGNESLLKARALVAFHQGIYPELYSILESHSFESANHPLLQQL        185
sp|Q8N196|SIX5_HUMAN            SRFLGALP----PAERLRGSDPVLRARALVAFQRGEYAELYRLLESRPFPAAHHAFLQDL        163
sp|O95475|SIX6_HUMAN            GRFLWSLPVAPAACEALNKNESVLRARAIVAFHGGNYRELYHILENHKFTKESHAKLQAL        90
sp|Q27350|SO_DROME              GRFLWSLP----QCDKLQLNESVLKAKAVVAFHRGQYKELYRLLEHHHFSAQNHAKLQAL        180
                                .*** :**     .: :.  : :*:*:*:***: * : :** :** : *    *  ** :
                                                    R110G/Q/W       E125K
                                               V106G   | R112C W122R  | Y129C  K134R
                                                  \    | |         |  |   |    |
sp|Q15475|SIX1_HUMAN            WLKAHYVEAEKLRGRPLGAVGKYRVRRKFPLPRTIWDGEETSYCFKEKSRGVLREWYAHN        146
sp|Q9NPC8|SIX2_HUMAN            WLKAHYIEAEKLRGRPLGAVGKYRVRRKFPLPRSIWDGEETSYCFKEKSRSVLREWYAHN        146
sp|O95343|SIX3_HUMAN            WLEAHYQEAEKLRGRPLGPVDKYRVRKKFPLPRTIWDGEQKTHCFKERTRSLLREWYLQD        228
sp|Q9UIU6|SIX4_HUMAN            WYKARYTEAERARGRPLGAVDKYRLRRKFPLPRTIWDGEETVYCFKEKSRNALKELYKQN        245
sp|Q8N196|SIX5_HUMAN            YLRARYHEAERARGRALGAVDKYRLRKKFPLPKTIWDGEETVYCFKERSRAALKACYRGN        223
sp|O95475|SIX6_HUMAN            WLEAHYQEAEKLRGRPLGPVDKYRVRKKFPLPRTIWDGEQKTHCFKERTRHLLREWYLQD        150
sp|Q27350|SO_DROME              WLKAHYVEAEKLRGRPLGAVGKYRVRRKFPLPRTIWDGEETSYCFKEKSRSVLRDWYSHN        240
                                : .*:* ***: *** ** *.***:*:*****::*****:. :****::*  *:  *  :
                                                        K173N
                                  P151L            W171C  | N174Y/K
                                    |                  \  |/
sp|Q15475|SIX1_HUMAN            PYPSPREKRELAEATGLTTTQVSNWFKNRRQRDRAAEAKER--ENTENNNSSSNKQNQLS        204
sp|Q9NPC8|SIX2_HUMAN            PYPSPREKRELAEATGLTTTQVSNWFKNRRQRDRAAEAKER--ENNENSNSNSHN-----        199
sp|O95343|SIX3_HUMAN            PYPNPSKKRELAQATGLTPTQVGNWFKNRRQRDRAAAAKNR--LQHQAIGPSGMRS----        282
sp|Q9UIU6|SIX4_HUMAN            RYPSPAEKRHLAKITGLSLTQVSNWFKNRRQRDRNPSETQS--KSESDGNPSTEDESSK-        302
sp|Q8N196|SIX5_HUMAN            RYPTPDEKRRLATLTGLSLTQVSNWFKNRRQRDRTGAGGGAPCKSESDGNPTTEDESSRS        283
sp|O95475|SIX6_HUMAN            PYPNPSKKRELAQATGLTPTQVGNWFKNRRQRDRAAAAKNR--LQQQVLSQGSGRA----        204
sp|Q27350|SO_DROME              PYPSPREKRDLAEATGLTTTQVSNWFKNRRQRDRAAEHKDG--STDKQHLDSSSDS----        294
                                 **.* :** **  ***: ***.***********            .
                                                             D227Y
                                                               |
sp|Q15475|SIX1_HUMAN            PLE---GGKPLMSSS------EEEFSPPQSPDQNSV-----LLLQ---------------        235
sp|Q9NPC8|SIX2_HUMAN            PLN--GSGKSVLGSS------EDEKTPSGTPDHSSSSP--ALLLSP----------PPPG        239
sp|O95343|SIX3_HUMAN            -LA--EPGCPTHGSA-------ESPSTAASPTT--------------------------S        306
sp|Q9UIU6|SIX4_HUMAN            -------G-------------HEDLSPHPLSSSSDGITNLSLSSHMEPVYMQQIGNAKIS        342
sp|Q8N196|SIX5_HUMAN            PEDLERGAAPVSAEA---------------------AA-------QGSIFLAGTGPPAPC        315
sp|O95475|SIX6_HUMAN            -LR--AEGDG---TP-------EVLGVATSPAA--------------------------S        225
sp|Q27350|SO_DROME              EME--GSMLPSQSAQHQQQQQQQQHSPGNSSGNNNGLHQQQLQHVA----------AEQG        342

                                                          P249L
                                                            |
sp|Q15475|SIX1_HUMAN            ----GNMGHA--RS---SNY------SLPGLT------A--SQPSHG-------------        259
sp|Q9NPC8|SIX2_HUMAN            LPSLHSLGHP--PGPSAVPV------PVPGGG------------GAD-------------        266
sp|O95343|SIX3_HUMAN            VSS---LTER--ADTGTSILSVTSSDSECDV-----------------------------        332
sp|Q9UIU6|SIX4_HUMAN            LSSSGVLLNGSLVPASTSPVFLNGNSFIQGPSGVILNGLNVGNTQAVA--------LNPP        394
sp|Q8N196|SIX5_HUMAN            PASSSILVNGSFLAASGSPA------VLLNGGPVIINGLALGEASSLGPLLLTGGGGAPP        369
sp|O95475|SIX6_HUMAN            LS----------SKAATSAISITSSDSECDI-----------------------------        246
sp|Q27350|SO_DROME              LQH---HPHQ--PHPASNIANVAATKSSGGGGG---GGV--SAAAAAQ--------MQMP        384
```

Amino acids at which pathogenic missense variants in SIX1 have been identified, either described in this work (V17E, P151L) or previously reported, are highlighted in red. Previously reported missense variants as unlikely to play a pathogenic role (too common and/or predicted benign, as annotated in Supplementary Table S2) are highlighted in green. An asterisk (*) indicates positions which have a single, fully conserved residue, a colon (:) indicates conservation between amino acids with strongly similar properties, and a period (.) indicates conservation between amino acids with weakly similar properties.

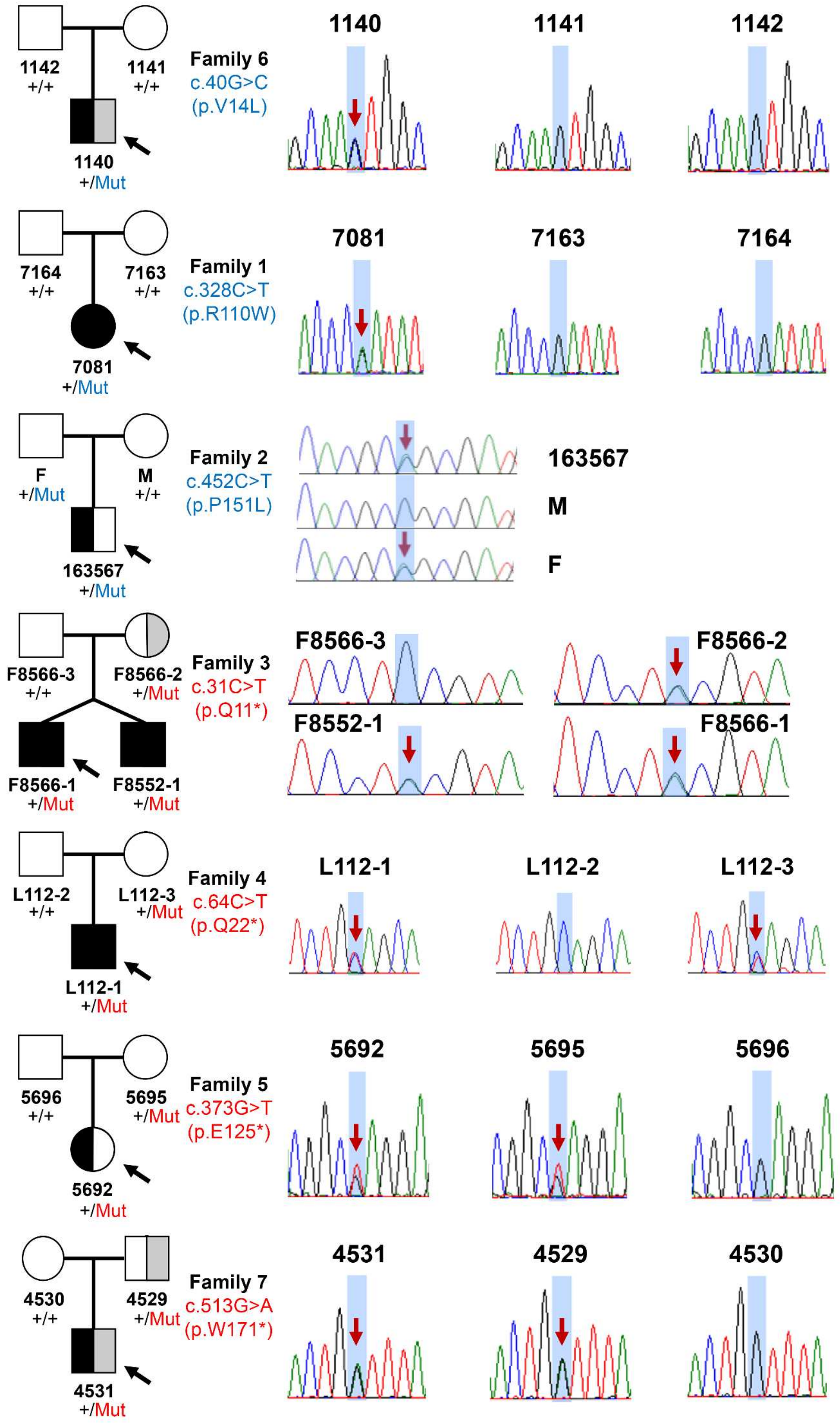

Figure S1. Dideoxy-sequence verification of *SIX1* variants

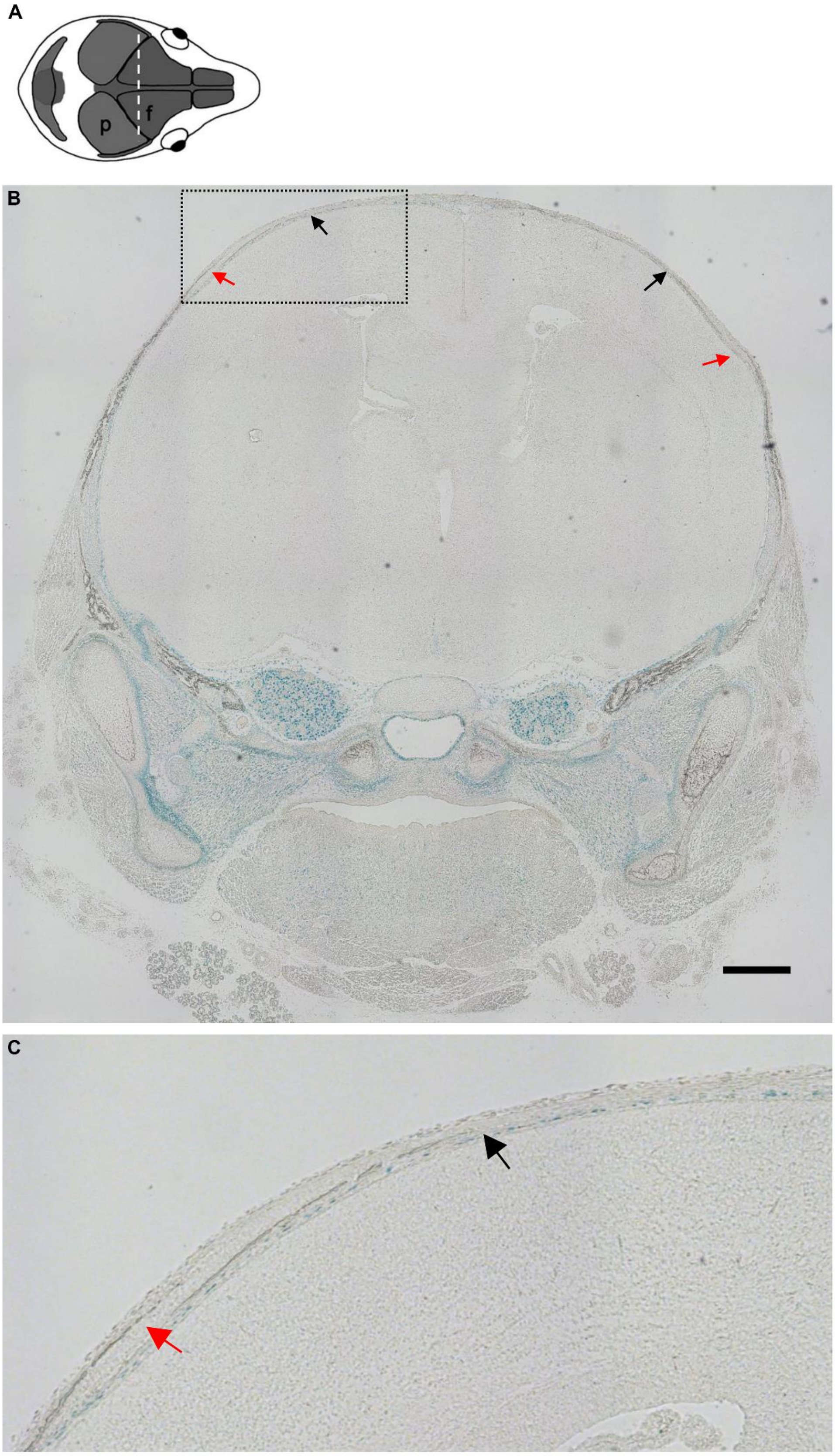
A
p
f
B
C

Figure S2. SIX1 expression revealed by X-gal staining in additional coronal section of E18.5 *Six1*$^{nLacZ/+}$ mouse head

(A) The section shown is anterior to that illustrated in figure 1E-F, so that the parietal and frontal bones, and the intervening coronal suture, are included in the section. **(B)** Black arrows indicate the superior margin of frontal bone and red arrows the coronal suture (parietal bone is outside the arrow point, frontal bone is underneath the arrowhead), and the dotted box (enlarged in **C**) shows the coronal suture. Blue nuclei (X-gal positive) indicate SIX1 expression. Scale bar: 500 µm. Figure part (A) adapted from Twigg and Wilkie[25] with permission.

References

1. Kochhar A, Orten DJ, Sorensen JL, et al. SIX1 mutation screening in 247 branchio-oto-renal syndrome families: a recurrent missense mutation associated with BOR. *Hum Mutat* 2008;29:565.
2. Mutai H, Suzuki N, Shimizu A, et al. Diverse spectrum of rare deafness genes underlies early-childhood hearing loss in Japanese patients: a cross-sectional, multi-center next-generation sequencing study. *Orphanet J Rare Dis* 2013;8:172.
3. Ruf RG, Xu PX, Silvius D, et al. SIX1 mutations cause branchio-oto-renal syndrome by disruption of EYA1-SIX1-DNA complexes. *Proc Natl Acad Sci USA* 2004;101:8090-8095.
4. Retterer K, Juusola J, Cho MT, et al. Clinical application of whole-exome sequencing across clinical indications. *Genet Med* 2016;18:696-704.
5. Iglesias A, Anyane-Yeboa K, Wynn J, et al. The usefulness of whole-exome sequencing in routine clinical practice. *Genet Med* 2014;16:922-931.
6. Patrick AN, Schiemann BJ, Yang K, Zhao R, Ford HL. Biochemical and functional characterization of six SIX1 Branchio-oto-renal syndrome mutations. *J Biol Chem* 2009;284:20781-20790.
7. Shah AM, Krohn P, Baxi AB, et al. Six1 proteins with human branchio-oto-renal mutations differentially affect cranial gene expression and otic development. *Dis Model Mech* 2020; 13:dmm043489.
8. Reese MG, Eeckman FH, Kulp D, Haussler D. Improved splice site detection in Genie. *J Comput Biol* 1997;4:311-323.
9. Patrick AN, Cabrera JH, Smith AL, Chen XS, Ford HL, Zhao R. Structure-function analyses of the human SIX1-EYA2 complex reveal insights into metastasis and BOR syndrome. *Nat Struct Mol Biol* 2013;20:447-453.
10. Calpena E, Cuellar A, Bala K, et al. SMAD6 variants in craniosynostosis: genotype and phenotype evaluation. *Genet Med* 2020; doi: 10.1038/s41436-020-0817-2.
11. Rentzsch P, Witten D, Cooper GM, Shendure J, Kircher M. CADD: predicting the deleteriousness of variants throughout the human genome. *Nucleic Acids Res* 2019;47:D886-D894.
12. Heidet L, Moriniere V, Henry C, et al. Targeted exome sequencing identifies PBX1 as involved in monogenic congenital anomalies of the kidney and urinary tract. *J Am Soc Nephrol* 2017;28:2901-2914.
13. Sanggaard KM, Rendtorff ND, Kjaer KW, et al. Branchio-oto-renal syndrome: detection of EYA1 and SIX1 mutations in five out of six Danish families by combining linkage, MLPA and sequencing analyses. *Eur J Hum Genet* 2007;15:1121-1131.
14. Mosrati MA, Hammami B, Rebeh IB, et al. A novel dominant mutation in SIX1, affecting a highly conserved residue, result in only auditory defects in humans. *Eur J Med Genet* 2011;54:e484-488.

Calpena E, *et al. J Med Genet* 2022; 59:165–169. doi: 10.1136/jmedgenet-2020-107459

15. Ito T, Noguchi Y, Yashima T, Kitamura K. SIX1 mutation associated with enlargement of the vestibular aqueduct in a patient with branchio-oto syndrome. *Laryngoscope* 2006;116:796-799.
16. Krug P, Moriniere V, Marlin S, et al. Mutation screening of the EYA1, SIX1, and SIX5 genes in a large cohort of patients harboring branchio-oto-renal syndrome calls into question the pathogenic role of SIX5 mutations. *Hum Mutat* 2011;32:183-190.
17. Sheppard S, Biswas S, Li MH, et al. Utility and limitations of exome sequencing as a genetic diagnostic tool for children with hearing loss. *Genet Med* 2018;20:1663-1676.
18. Plevova P, Paprskarova M, Tvrda P, Turska P, Slavkovsky R, Mrazkova E. STRC deletion is a frequent cause of slight to moderate congenital hearing impairment in the Czech Republic. *Otol Neurotol* 2017;38:e393-e400.
19. Unzaki A, Morisada N, Nozu K, et al. Clinically diverse phenotypes and genotypes of patients with branchio-oto-renal syndrome. *J Hum Genet* 2018;63:647-656.
20. Kari E, Llaci L, Go JL, et al. A de novo SIX1 variant in a patient with a rare nonsyndromic cochleovestibular nerve abnormality, cochlear hypoplasia, and bilateral sensorineural hearing loss. *Mol Genet Genomic Med* 2019;7:e995.
21. Fernández-Alvarez P, Cueto-González A, Vendrell Bayona T, et al. Abstracts from the 50th European Society of Human Genetics Conference: Electronic Poster E-P02.02 Novel mutation in SIX1 in a girl with branchiotic syndrome. *Eur J Hum Genet* 2019;26:820-1023.
22. Carminho-Rodrigues T, Guney M, Carvalho B, et al. Abstracts from the 50th European Society of Human Genetics Conference: Poster P11.104D. A novel familial SIX1 mutation with incomplete penetrance in Branchio-otic Syndrome. *Eur J Hum Genet* 2019;26:113-819.
23. Li B, Xu L, Hong N, Chen S, Xu R. In Silico Analyses Reveal the Relationship Between SIX1/EYA1 Mutations and Conotruncal Heart Defects. *Pediatr Cardiol* 2018;39:176-182.
24. Weber S, Moriniere V, Knuppel T, et al. Prevalence of mutations in renal developmental genes in children with renal hypodysplasia: results of the ESCAPE study. *J Am Soc Nephrol* 2006;17:2864-2870.
25. Twigg SRF, Wilkie AOM. A Genetic-pathophysiological framework for craniosynostosis. *Am J Hum Genet* 2015;97:359-377.
